# Supplementary material for: Ligand-induced transmembrane conformational coupling in monomeric EGFR
Source: Nat Commun. 2022 Jul 6;13:3709. doi: 10.1038/s41467-022-31299-z (PMC9259572; doi:10.1038/s41467-022-31299-z)
Supplement: Supplementary file 1 — Supplementary Information [file 41467_2022_31299_MOESM1_ESM.pdf]

Supplementary Information: Ligand-induced transmembrane conformational coupling in the monomeric epidermal growth factor receptor

G.S. Schlau-Cohen *et al.*

This document contains the following supporting information:

Supplementary Figures 1 – 29

Supplementary Tables 1 – 17

Supplementary References (1 - 9)

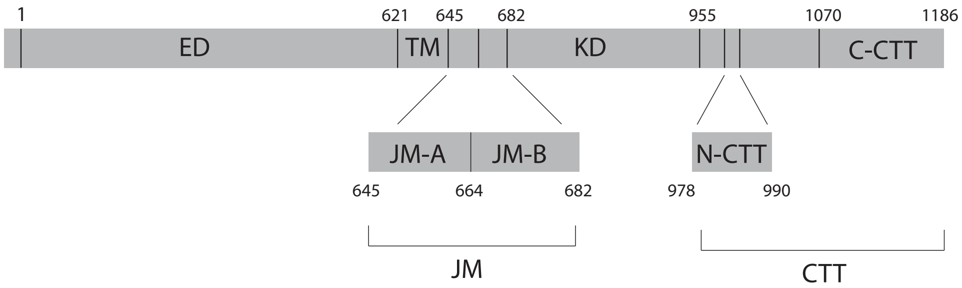

Supplementary Figure 1. Domains of EGFR. EGFR consists of a 621-amino acid extracellular region (ED), a 24-amino acid transmembrane-spanning domain (TM), and an intracellular region, which is a 37-amino acid juxtamembrane domain (JM), a 273-amino acid kinase domain (KD) and a 231-amino acid C-terminal tail (CTT). The JM is further divided into juxtamembrane-A (JM-A) and juxtamembrane-B (JM-B) domains. Residues 978–990 are defined as N-terminal portion of the CTT (N-CTT) and residues 1070–1186 are defined as the C-terminal portion of the CTT (C-CTT). Residue numbering corresponds to EGFR excluding the signal sequence.

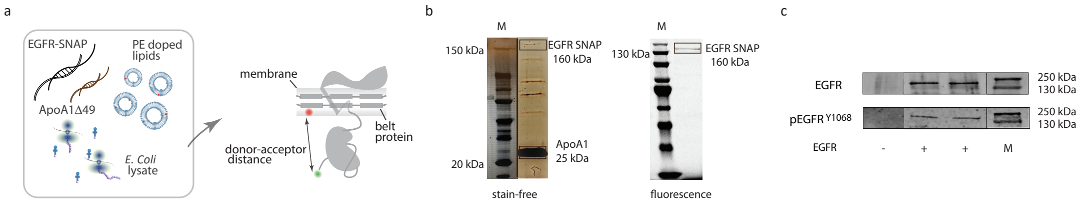

Supplementary Figure 2. Production and characterization of full-length EGFR in nanodiscs. (a) Cell-free reaction for the production of EGFR and nanodisc belt protein. Codon optimized DNA of the receptor protein (EGFR-SNAP) and the belt protein (ApoA1Δ49) are incubated overnight together with lipid vesicles and $E.\ Coli$ lysate at 25° C. (b) Stain-free (left) and fluorescence (right) gel images of the His-tag purified sample show the presence of ApoA1 at 25 kDa and full-length EGFR at 160 kDa, which implies successful EGFR production and insertion into nanodiscs. The presence of the EGFR band alone in the fluorescence gel image indicates successful and specific labeling. Lane M is protein marker. The gel images reported are representative of eight biological replicates. (c) Western blots were performed on labelled EGFR in nanodiscs. Anti-EGFR Western blots (top) and anti-phosphotyrosine Western blots (bottom) tested the presence of EGFR and its ability to undergo tyrosine phosphorylation, respectively. Bands were observed in both blots when the EGFR plasmid was present, consistent with previous experiments on similar preparations. Lane M is protein marker. The blots reported are representative of four biological replicates.[1,2] Source data are provided as a Source Data file.

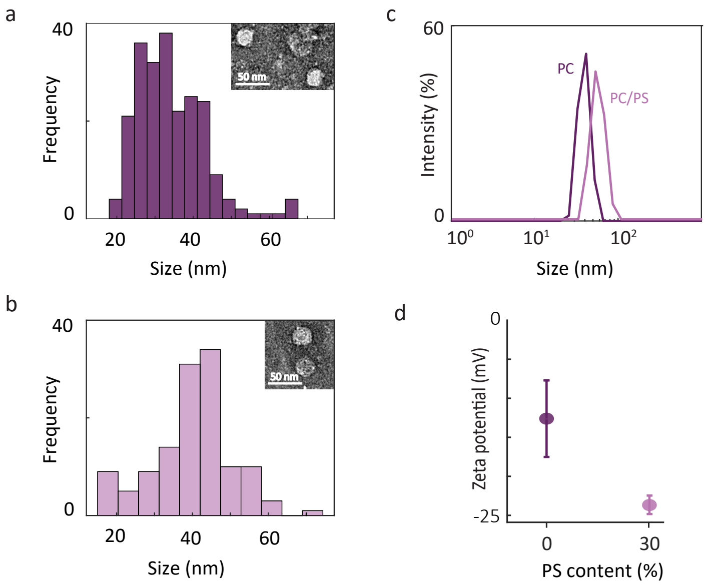

Supplementary Figure 3. Characterization of EGFR-containing nanodiscs. (a) Size distribution of EGFR in DMPC nanodiscs from negative stain transmission electron microscopy (TEM). The circles in the images (inset) show formation of DMPC nanodiscs. The mean disc diameter is 34.1 $\pm$ 8.8 nm (N = 224). (b) Size distribution of EGFR in POPC-POPS (70% POPC/30% POPS) nanodiscs from negative stain TEM. The circles in the images (inset) show formation of POPC-POPS nanodiscs. The mean disc diameter is 40.1 $\pm$ 11.0 nm (N = 126). (c) Dynamic light scattering (DLS) of EGFR in DMPC nanodiscs in PBS buffer indicates $\sim$37 nm average size, consistent with the TEM results and the FCS curves shown in the Fig. 3a in the main text. DLS of EGFR in POPC-POPS nanodiscs in PBS buffer indicates $\sim$53 nm average size, similar to the TEM results. (d) Maximum values from the zeta potential distributions for EGFR in DMPC (dark purple) and POPC-POPS (light purple) nanodiscs distributions in Fig. 2c of main text. Data are presented as mean values $\pm$ standard deviation. Zeta potential of EGFR in DMPC nanodiscs indicates a charge of -12.61 $\pm$ 4.81 mV (maximum: -8.5 mV; minimum: -19.8 mV) and zeta potential of EGFR in POPC-POPS nanodiscs indicates a charge of -23.68 $\pm$ 1.24 mV (maximum: -22.7 mV; minimum: -35.6 mV). Standard deviation is estimated from five technical replicates. Source data are provided as a Source Data file.

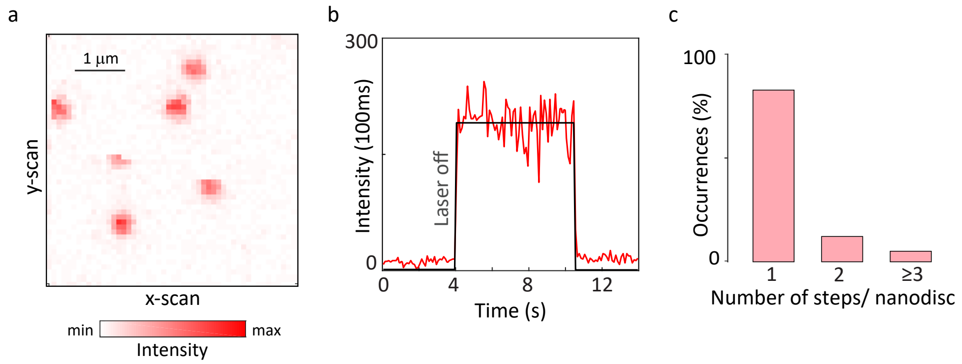

Supplementary Figure 4. Photobleaching analysis of Cy5 acceptor molecules in nanodiscs. (a) Confocal fluorescence image of immobilized constructs of EGFR in nanodiscs containing a labelled Cy5 lipid using 640 nm excitation. (b) Representative intensity time trace from a single construct. The number of detected photons for each 100 ms interval was calculated and used to generate a fluorescence intensity trace (red) with the average intensity for the emissive period overlaid (black). (c) Number of step-wise intensity changes per time trace (N=139). Most molecules (∼85%) exhibited a single-step intensity trajectory indicating the presence of single acceptor molecules.

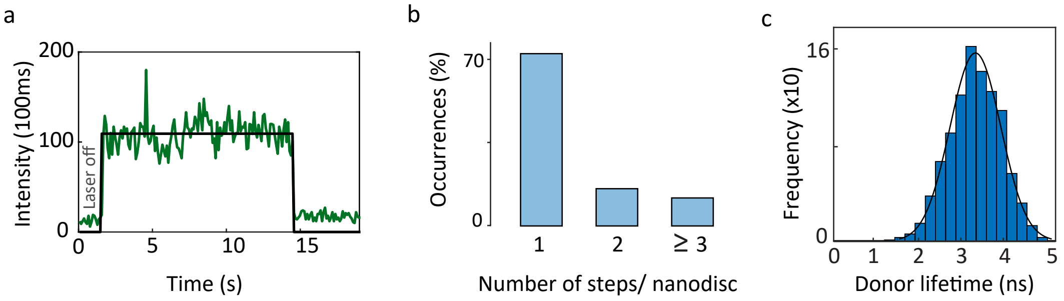

Supplementary Figure 5. Photobleaching analysis and lifetime distribution of donor-only constructs. (a) Representative time trace from single-molecule fluorescence of monomeric donor-only EGFR in nanodiscs showing single-step photobleaching. The number of detected photons for each 100 ms interval was calculated and used to generate a fluorescence intensity trace (green) with the average intensity overlaid (black). (b) Number of step-wise intensity changes per time trace (N=105). The majority of molecules (∼75%) exhibit single-step photobleaching, indicating primarily a single EGFR protein per nanodisc as in previous work.[2] (c) The fluorescence lifetime distribution for the donor-only construct with a median at 3.32 ns. Source data are provided as a Source Data file.

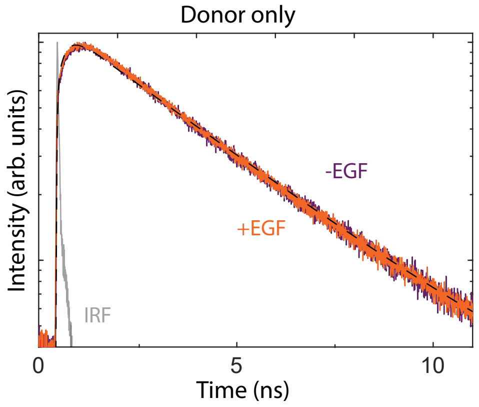

Supplementary Figure 6. Ensemble lifetime measurements of snap surface 594. Ensemble time-correlated single photon counting measurements were performed for the donor dye, snap surface 594 (free dye in 1× PBS, pH 7.4), in the absence (purple) and presence (orange) of EGF. The instrument response function (IRF) is shown in gray. For both decay traces, a mono-exponential fit (dashed line) found a 3.2 ns lifetime, indicating no photophysical perturbations were induced by EGF. Source data are provided as a Source Data file.

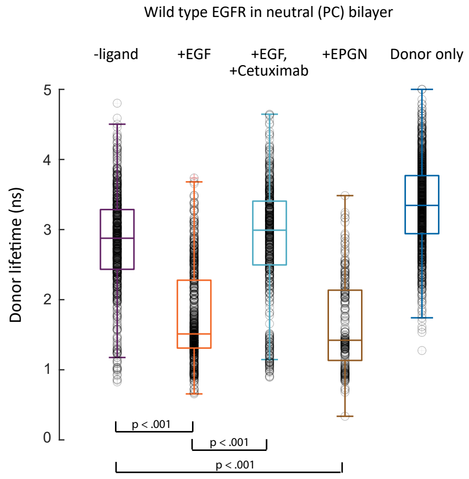

Supplementary Figure 7. Box plots of the donor lifetime distributions in a neutral lipid bilayer. Distributions of the donor lifetime from the histograms in Fig. 2a of main text are represented as box plots. One-way ANOVA was performed to obtain the P-values (exact P-values mentioned in Supplementary Table 10). No adjustments were made for multiple comparisons. The donor-only lifetime distribution showed a significant difference compared to other samples (P<0.001). A narrow lifetime distribution with a median of 3.32 ns was found for the donor-only sample. The median value was used as the reference value for calculations of the donor-acceptor distances for all smFRET measurements on WT EGFR in DMPC. Median, maximum, minimum, whiskers and quartiles of the distributions are reported in Supplementary Table 11. The number of data points in the smFRET lifetime distributions are from two to four biological replicates for each sample and reported in Supplementary Table 13. Source data are provided as a Source Data file.

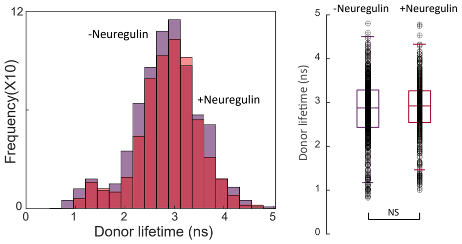

Supplementary Figure 8. Lifetime distributions for EGFR in nanodiscs in the presence of neuregulin. Neuregulin is a structurally similar ligand of the EGF family that does not bind EGFR.[3] smFRET measurements were performed on labelled EGFR in nanodiscs in the presence of neuregulin 1 μM and the donor lifetime distribution (N=69) was constructed. (left) The distribution is overlaid with the distribution in the absence of ligand (median = 2.88 ns). The lifetime distribution in the absence of ligand is taken from main text (-EGF, Fig. 2a, top). (right) The distribution in the presence of neuregulin (median = 2.92 ns) is statistically similar to the distribution in the absence of ligand (P = 0.2654), indicating the EGF-dependence of the conformational changes reported in the main text. NS, not significant. Median, maximum, minimum, whiskers and quartiles of the distributions are reported in Supplementary Table 11. Source data are provided as a Source Data file.

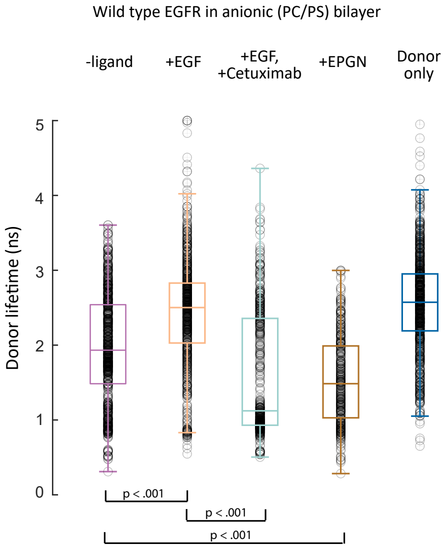

Supplementary Figure 9. Box plots of the donor lifetime distributions in a partially anionic bilayer. Distributions of donor lifetime from the histograms in Fig. 2d of main text are represented as box plots. One-way ANOVA was performed to obtain the P-values (exact P-values mentioned in Supplementary Table 10). No adjustments were made for multiple comparisons. The donor-only lifetime distribution showed a significant difference compared to other samples ($P<0.001$). A narrow lifetime distribution with a median of 2.6 ns was found for the donor-only sample. The median value was used as the reference value for calculations of the donor-acceptor distances for all smFRET measurements on WT EGFR in POPC-POPS. Median, maximum, minimum, whiskers and quartiles of the distributions are reported in Supplementary Table 12. The number of data points in the smFRET lifetime distributions are from two to four biological replicates for each sample and reported in Supplementary Table 13. Source data are provided as a Source Data file.

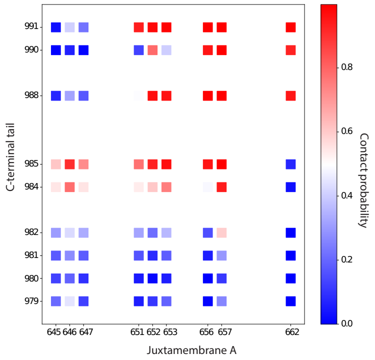

Supplementary Figure 10. Critical residues for the electrostatic interaction between the JM domain and the CTT. The contact probability between the positively charged residues of the JM-A region (residues 645–664) and the negatively-charged residues of the CTT (residues 978–995) was determined using the all-atom simulation of active EGFR. The contact probability was calculated as the fraction of time each pair of residues is closer than 2.0 nm ($\sim$3 times the Debye-Huckel screening length). The residues in the C-terminal half of the CTT have a higher contact probability with the residues in the C-terminal half of the JM-A region (upper right). See text *Section: Atomistic Simulations with the CHARMM Force Field* for simulation details.

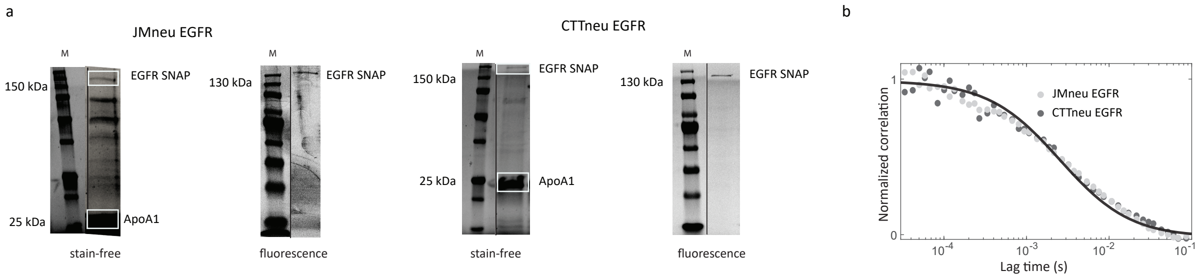

Supplementary Figure 11. Characterization of JMneu EGFR and CTTneu EGFR produced via **cell-free reaction.** (a) Stain-free and fluorescence gel images show the presence of ApoA1Δ49 at 25 kDa and full-length EGFR at 160 kDa in the His-tag purified sample, which implies successful synthesis and insertion into nanodiscs in cell-free production of JMneu EGFR and CTTneu EGFR. Lane M is protein marker. Source data are provided as a Source Data file. (b) Fluorescence correlation spectroscopy (FCS) curves of the EGFR mutants in nanodiscs (dots) diffusing through the confocal volume. Solid line represents the fit of the FCS curve for JMneu EGFR. The diffusion timescales (2.54 ms for JMneu EGFR and 2.60 ms for CTTneu EGFR) were comparable to that of WT EGFR (2.2 ms), which shows that constructs of comparable sizes were formed. The results reported are representative of three biological replicates.

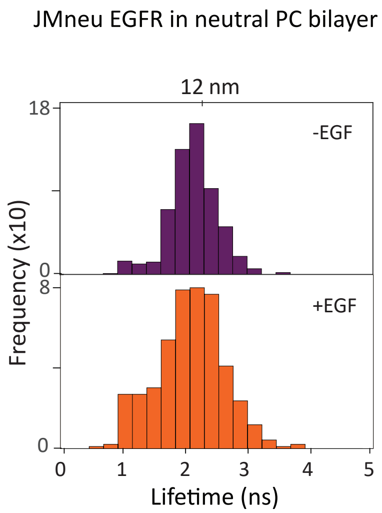

Supplementary Figure 12. smFRET measurements of JMneu EGFR. smFRET donor fluorescence lifetime histograms for JMneu EGFR in a neutral bilayer. Both sets of histograms are statistically similar in the absence (top) and presence (bottom) of EGF (Supplementary Figure 14), indicating the EGF-dependent intracellular conformational change was lost upon neutralization of the JM. Median, maximum, minimum, whiskers and quartiles of the distributions are reported in Supplementary Table 11. Source data are provided as a Source Data file.

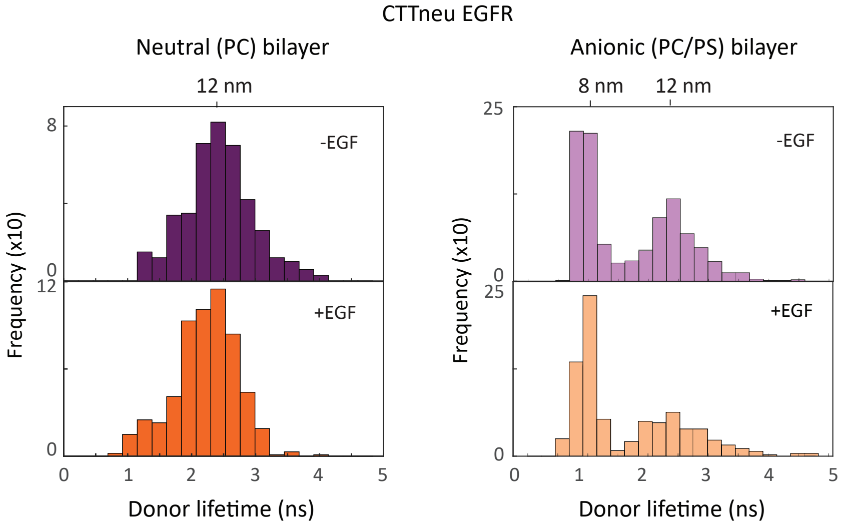

Supplementary Figure 13. smFRET measurements of CTTneu EGFR. smFRET donor fluorescence lifetime histograms for CTTneu EGFR in a (left) neutral bilayer and (right) partially anionic lipid bilayer. Both sets of histograms are statistically similar in the absence (top) and presence (bottom) of EGF (Supplementary Figure 15), indicating the EGF-dependent intracellular conformational change was lost upon neutralization of the CTT. Median, maximum, minimum, whiskers and quartiles of the distributions are reported in Supplementary Table 11, 12. Source data are provided as a Source Data file.

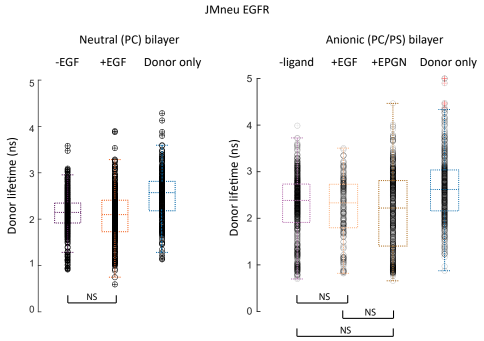

Supplementary Figure 14. Box plots of the donor lifetime distributions for the JMneu EGFR mutant in a neutral and partially anionic lipid bilayer. Distributions of donor lifetime from the histograms in Fig. 3c of main text and Supplementary Figure 12 are represented as box plots. One-way ANOVA was performed to obtain the P-values (exact P-values mentioned in Supplementary Table 10). No adjustments were made for multiple comparisons. The donor-only lifetime distribution showed a significant difference compared to other samples (P< 0.001). The donor-only lifetime distribution depends on the local electrostatic environment[4] and so was individually measured for each mutant and for each lipid environment. The medians of the donor-only distributions (2.57 ns in neutral bilayer, and 2.61 ns in partially anionic bilayer) were used as references to calculate the donor-acceptor distances for the smFRET measurements on JMneu EGFR. Median, maximum, minimum, whiskers and quartiles of the distributions are reported in Supplementary Table 11, 12. The number of data points in the smFRET lifetime distributions are from two to four biological replicates for each sample and reported in Supplementary Table 13. Source data are provided as a Source Data file.

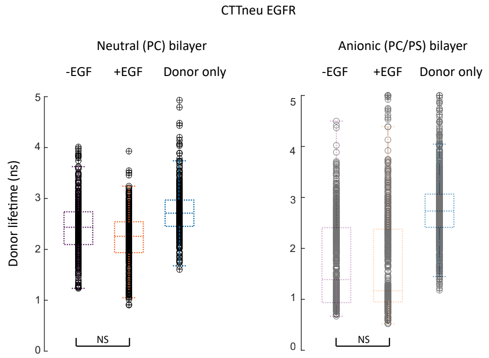

Supplementary Figure 15. Box plots of the donor lifetime distributions for the CTTneu EGFR mutant in a neutral and partially anionic lipid bilayer. Distributions of donor lifetime from the histograms in Supplementary Figure 13 are represented as box plots. One-way ANOVA was performed to obtain the P-values (exact P-values mentioned in Supplementary Table 10). No adjustments were made for multiple comparisons. The donor-only lifetime distribution showed a significant difference compared to other samples (P< 0.001). The donor-only lifetime distribution depends on the local electrostatic environment[4] and so was individually measured for each mutant and for each lipid environment. The medians of the donor-only distributions (2.71 ns in neutral bilayer, and 2.73 ns in partially anionic bilayer) were used as references to calculate the donor-acceptor distances for the smFRET measurements on CTTneu EGFR. Median, maximum, minimum, whiskers and quartiles of the distributions are reported in Supplementary Table 11, 12. The number of data points in the smFRET lifetime distributions are from two to four biological replicates for each sample and reported in Supplementary Table 13. Source data are provided as a Source Data file.

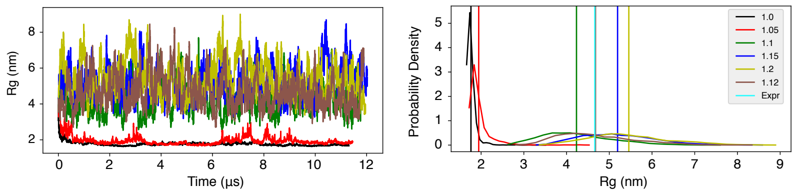

Supplementary Figure 16. Adjusting the Martini force field to improve its performance in modeling the CTT portions of the EGFR (residues 961–1186). The initial configuration of these simulations was prepared with the CHARMM-gui toolkit[5] by solvating the CTT with water molecules and 0.15 M neutralizing ions (NaCl). The CTT structure was built using Modeller.[6] We then carried out independent constant temperature (303K), constant volume simulations with the Lennard-Jones interactions between protein atoms and water molecules scaled by a factor of 1.0, 1.05, 1.1, 1.12, 1.15, and 1.2. Approximately 12 μs of simulations were performed, and the CTT radius of gyration (R_g) as a function of time along different trajectories is shown on the left. The corresponding probability distributions of Rg are provided on the right. The scaling factor of 1.12 (brown) best reproduces the experimental R_g value (cyan) of 4.66 nm. See text *Section: Coarse-grained, Explicit-solvent Simulations with the MARTINI Force Field* for additional simulation details. Source data are provided as a Source Data file.

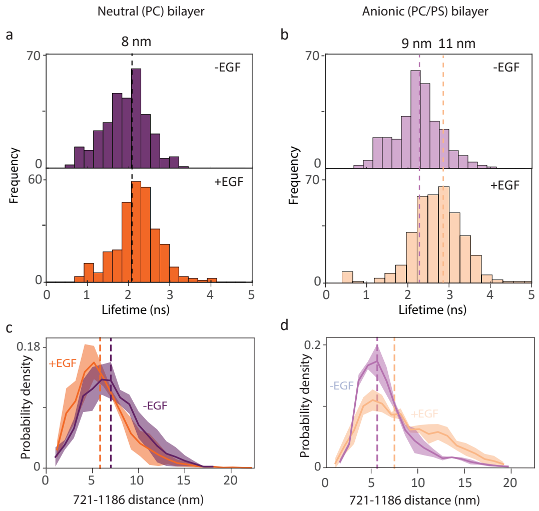

Supplementary Figure 17. Comparison of the distance between the ATP binding site and the C-terminus of EGFR from smFRET experiments and simulations. To further establish the correspondence between the results obtained from experiments and simulations, the distance between the ATP binding site (residue 721) and C-terminus of the protein (residue 1186) was measured using smFRET experiments with a fluorescently-labeled ATP analog and extracted from the simulations for both the neutral and partially anionic lipid bilayers. The results from the smFRET measurements were used to build histograms of the donor lifetime for WT EGFR. (a) In the neutral bilayer, the distribution peaked at ∼8 nm in both the absence (top, purple) and presence (bottom, orange) of EGF. (b) In the partially anionic bilayer, the donor lifetime distributions peaked at 10.9 nm in the presence of EGF and 9 nm in the absence of EGF. The distances between residues 721 and 1186 were calculated from the molecular dynamics trajectories of the simulated conformations in both lipid bilayers and used to generate probability distributions for active (+EGF, orange) and inactive (-EGF, purple) EGFR. (c) In the neutral bilayer, the medians were similar at only ∼1 nm apart whereas (d) in the partially anionic bilayer, they were separated by ∼2 nm, consistent with the experimental trends. The shaded regions represent the standard deviation estimated with block averaging. The medians for all distributions are given in Supplementary Table 17. Source data are provided as a Source Data file.

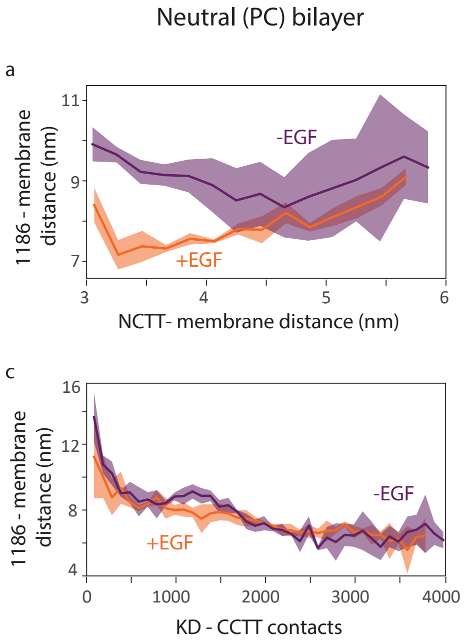

Supplementary Figure 18. The distances between the membrane and the C-terminus correlate with two collective conformational variables. The measured smFRET distance is approximated in the simulations by the distance between the z center of the mass of the lipid bilayer and residue 1186. (a, b) The distance between the bilayer and residue 1186 correlates positively with the distance between the z center of the mass of the lipid bilayer and the N-terminal portion of the tail (NCTT, residues 978–990) for simulations with active (+EGF, orange) and inactive (-EGF, purple) EGFR embedded in neutral (a) or partially negative (b) bilayer. (c, d) The distance between the lipid bilayer and residue 1186 correlates negatively with the contacts formed between KD and the C-terminal portion of the tail (CCTT, residues 1070–1186) in the same simulations. Two atoms were recognized as in contact if they were separated by less than 8Å. The shaded regions represent the standard deviation estimated with block averaging. Source data are provided as a Source Data file.

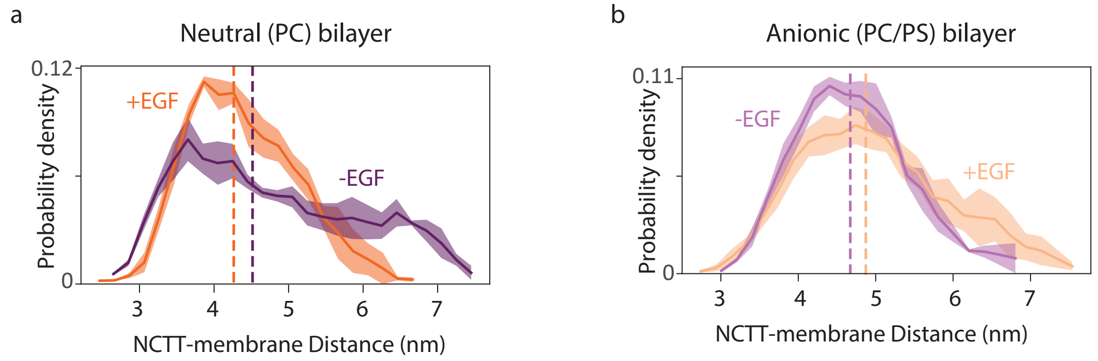

Supplementary Figure 19. Probability distributions for the distance between the lipid bilayer and the N-terminal portion of the CTT (NCTT). The distances between the z center of mass for the lipid bilayer and for the NCTT (residues 978–990) were calculated using simulations with active (+EGF, orange) and inactive (-EGF, purple) EGFR embedded in neutral (a) or partially negative (b) bilayer. The median values of the distributions are indicated by dashed vertical lines (PC bilayer: 4.52 nm (-EGF) and 4.26 nm (+EGF); PC-PS bilayer: 4.67 nm (-EGF) and 4.87 nm (+EGF)). The shaded regions represent the standard deviation estimated with block averaging. The distance reaches the smallest and largest values for the active EGFR in the neutral and anionic membrane, respectively, which reflects the conformational compaction or expansion. Source data are provided as a Source Data file.

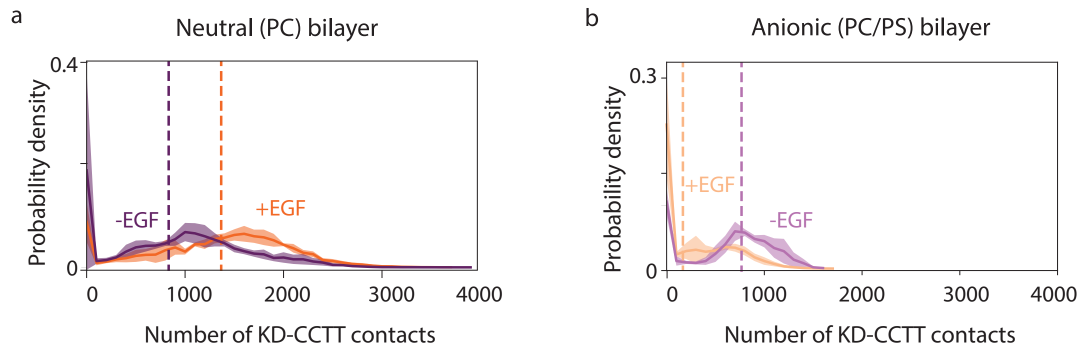

Supplementary Figure 20. Probability distributions for the contacts between the KD and the C-terminal portion of the CTT (CCTT). The number of contacts were calculated between the KD (residues 682–954) and the CCTT (residues 1070–1186) in simulations with active (+EGF, orange) and inactive (-EGF, purple) EGFR embedded in neutral (a) or partially anionic (b) bilayer. Two atoms were recognized as in contact if their separation was less than 8Å. The median values of the distributions are indicated by dashed vertical lines (PC bilayer: 833 (-EGF) and 1367 (+EGF); PC-PS bilayer: 767 (-EGF) and 167 (+EGF)). The shaded regions represent the standard deviation estimated with block averaging. The large number of contacts for active EGFR in the neutral membrane contributes to the conformational compaction. Source data are provided as a Source Data file.

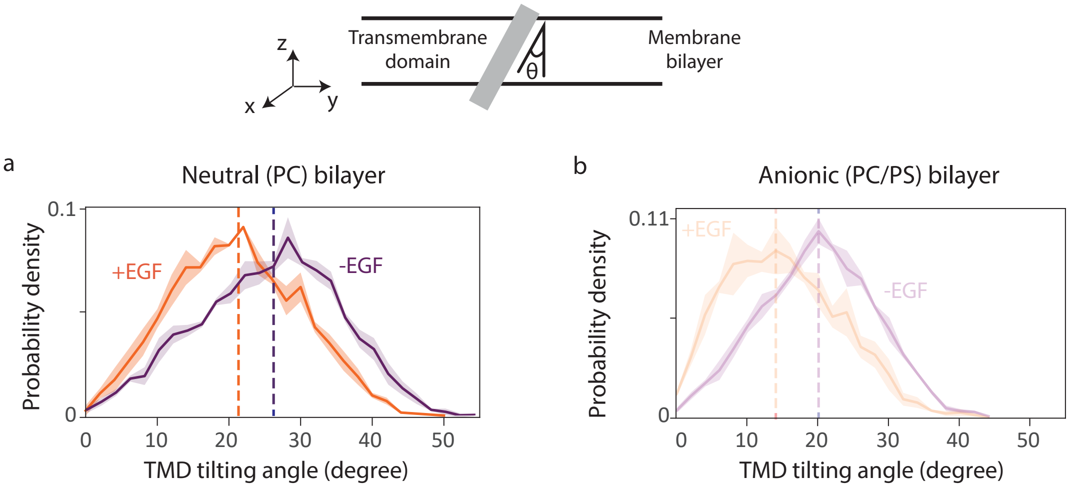

Supplementary Figure 21. Probability distributions of the TM domain tilting angle. The TM domain tilting angle (illustration in top panel) was extracted from simulations with active (+EGF, orange) and inactive (-EGF, purple) EGFR embedded in neutral (a) or partially anionic (b) lipid bilayer. The membrane bilayer stays parallel to the x-y plane throughout the simulations and so the TM domain tiling angle was quantified by measuring the angle θ between the TM helix and the z-axis. The vector along the TM helix was defined using the center of mass coordinates of the N- and C-terminal residues, with residue ID 622 and 644. The median values of the distributions are indicated by dashed vertical lines (PC bilayer: 26.2 degree (-EGF) and 21.3 degree (+EGF); PC-PS bilayer: 20.1 degree (-EGF) and 14.8 degree (+EGF)). The shaded regions represent the standard deviation estimated with block averaging. Source data are provided as a Source Data file.

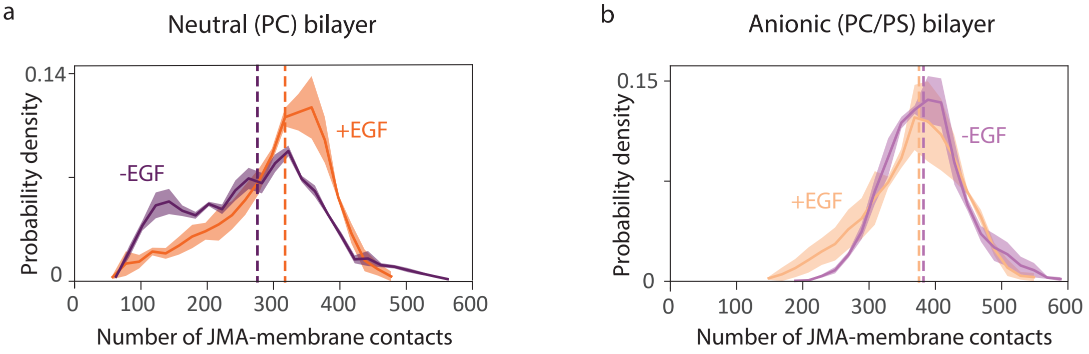

Supplementary Figure 22. Probability distributions of the contacts formed between the JM-A region and lipids. The number of contacts was calculated between the JM-A region (residues 645–664) and the lipid bilayer from simulations of active (+EGF, orange) and inactive (-EGF, purple) EGFR embedded in neutral (a) or partially negative (b) bilayer. Two atoms were defined as in contact if their separation was less than 8Å. The median values of the distributions are indicated by dashed vertical lines (PC bilayer: 276 (-EGF) and 317 (+EGF); PC-PS bilayer: 382 (-EGF) and 375 (+EGF)). The shaded regions represent the standard deviation estimated with block averaging. The contacts reflect the degree to which the JM-A embeds into the membrane, which is most significant for inactive EGFR in the anionic membrane. Source data are provided as a Source Data file.

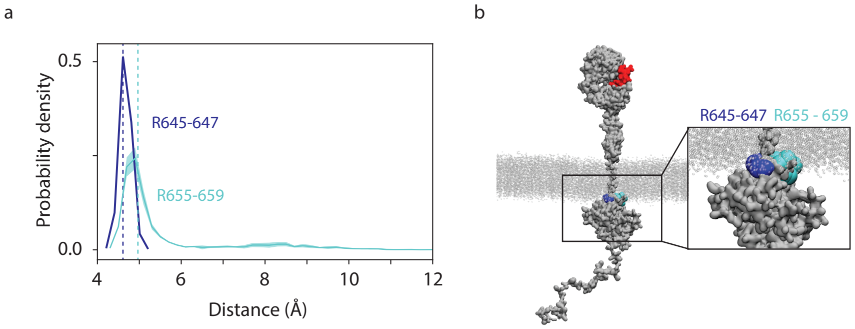

Supplementary Figure 23. Close contacts between JMA and the membrane revealed by coarse-grained simulations. (a) Probability distributions of the minimum distance between two regions of the JMA domain (R645-647, R655-659) and the phosphate groups of the inner leaflet of the bilayer. The median values of the distributions are indicated by vertical lines R645-647 minimal distance: 4.61 Å; R655-659 minimal distance: 4.97 Å. The shaded regions represent the standard deviation estimated with block averaging. Source data are provided as a Source Data file. (b) A representative structure of the ligand-bound EGFR from simulations with the POPC-POPS bilayer highlighting the interactions between the JMA domain (R645-647 (blue), R655-659 (cyan)) and the phosphate groups. The inset provides a closer look of the contacts.

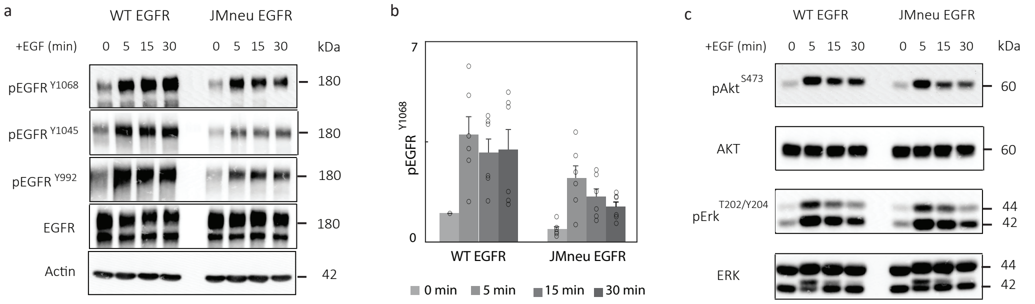

Supplementary Figure 24. Cellular experiments with JMneu EGFR show reduced phosphorylation. (a) Expression of phosphorylated EGFR (pEGFRY1068, pEGFRY1045, pEGFRY992) and total EGFR at 0, 5, 15, and 30 minutes of 100 ng/mL EGF stimulation in CHO cells transfected with WT EGFR and JMneu EGFR. Actin was used as a loading control. (b) Relative expression of pEGFRY1068 was determined by western blot quantification. The values were normalized to pEGFRY1068 before EGF stimulation (time 0) of the wild-type EGFR transfected cells (Supplementary Table 5). Data are presented as mean values ± SEM from six independent biological replicates (circles); average, SEM reported in Supplementary Table 15. Two-tailed, nonparametric, paired t-test was performed to obtain the P-values (exact P-values mentioned in Supplementary Table 14). No adjustments were made for multiple comparisons. (c) The expression level was quantified by Western blot for phosphorylated Akt (pAktS473), phosphorylated Erk (pErkT202/Y204) and total AKT, ERK at 0, 5, 15, and 30 minutes of 100 ng/mL EGF stimulation in CHO cells transfected with WT or JMneu EGFR. The blots reported are representative of seven independent biological replicates. Source data are provided as a Source Data file.

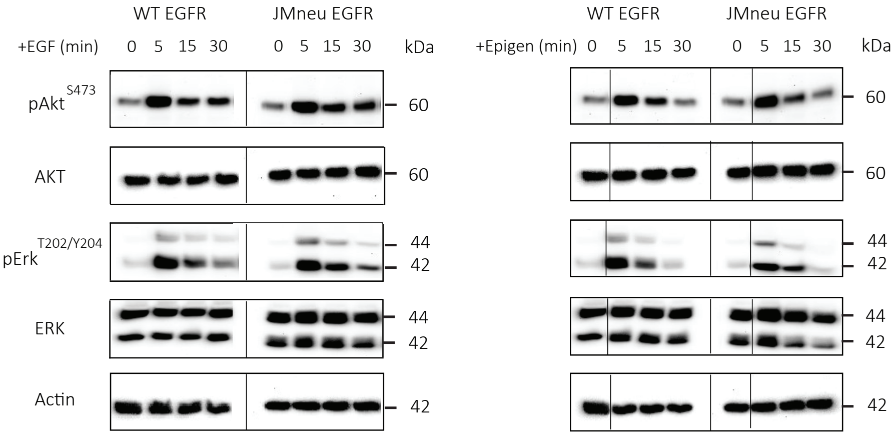

Supplementary Figure 25. Cellular experiments with JMneu EGFR show reduced signaling. The expression level was quantified by Western blot for phosphorylated Akt (pAktS473), phosphorylated Erk (pErkT202/Y204) and total AKT, ERK at 0, 5, 15, and 30 minutes of 100 ng/mL EGF (left) and 300 ng/mL epigen (right) stimulation in CHO cells transfected with WT or JMneu EGFR. Actin was used as a loading control. The blots reported are representative of six independent biological replicates. Black line delineates boundary between gels. Source data are provided as a Source Data file.

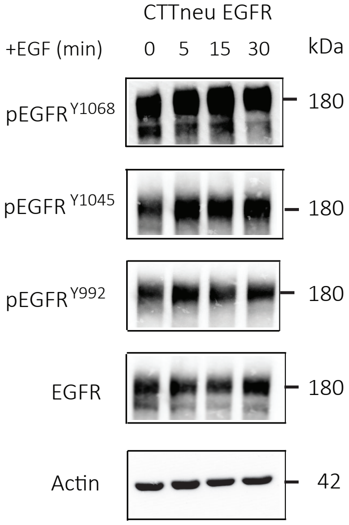

Supplementary Figure 26. Cellular experiments with CTTneu EGFR show high basal phosphorylation. The expression level was quantified by Western blot for phosphorylated EGFR (pEGFRY1068, pEGFRY1045, pEGFRY992) and total EGFR at 0, 5, 15, and 30 minutes of 100 ng/mL EGF stimulation in CHO cells transfected with CTTneu EGFR. Actin was used as a loading control. The blots reported are representative of seven independent biological replicates. Source data are provided as a Source Data file.

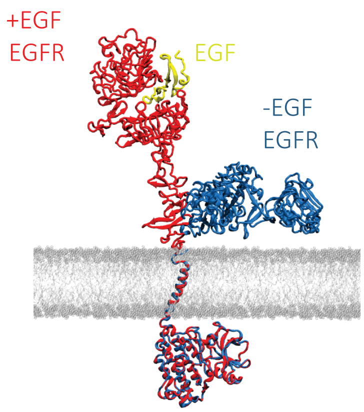

Supplementary Figure 27. Structural comparison between the ligand bound (+EGF, red) and unbound (-EGF, blue) EGFR. The two structures were aligned based on the intracellular kinase domain. The disordered CTT is not resolved in the crystal structure and is not shown in this figure. The lipid bilayer is shown in gray. These modeled structures were used to initialize our simulations with the CHARMM36m force field (CTT excluded) and the MARTINI force field (CTT included). The extracellular domain in the +EGF structure adopts a more upright configuration than the -EGF structure. The transmembrane and intracellular domains are the same between these two structures by construction.

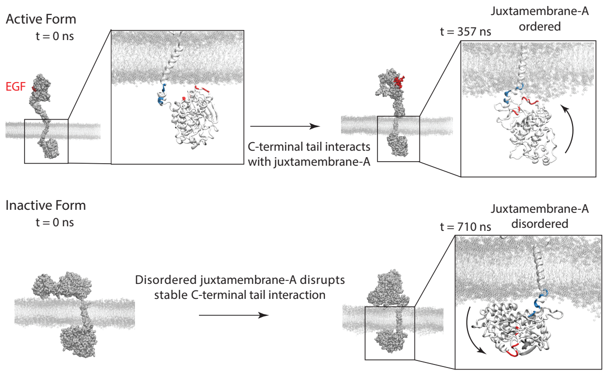

Supplementary Figure 28. Representative configurations obtained from all-atom explicit-solvent simulations of the active and inactive (chimera) EGFR with a neutral DMPC bilayer. See text *Atomistic Simulations with the CHARMM Force Field* for simulation details. Initial configurations of the two simulations constructed from structures reported in[7] are shown on the left. The juxtamembrane-A (JMA) domain formed close contacts with the N-terminal portion of the EGFR tail (NCTT) as a result of electrostatic interactions in the active simulation, but stayed far apart from each other in the inactive one. The charged residues are colored in blue for positive and red for negative in the insets. Because JMA is located near the membrane, its interaction with NCTT brought the EGFR tail close to the membrane as well. A similar mechanism with smaller NCTT-membrane distance was observed in the MARTINI simulations reported in the main text as well.

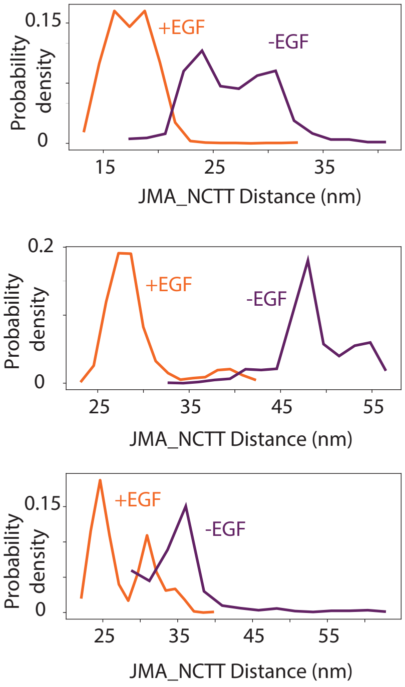

Supplementary Figure 29. Distributions of JMA-NCTT distance from three-independent all-atom simulations. Details of these simulations are provided in the Section: *Atomistic Simulations with the CHARMM Force Field*. All simulations support a closer distance between the NCTT and JMA in the +EGF EGFR. Source data are provided as a Source Data file.

[EGFR]	[Y1068]	Increase in diffusion time	Conclusions
Experiments at 550 nm, monitoring labeled EGFR			
1 nM (EGFR nanodiscs)	0 nM	-	-
1 nM (EGFR nanodiscs)	0.25 nM	20.4%	Antibody is bound to pY1068.
Experiments at 640 nm, monitoring labelled antibody			
0 nM (empty nanodiscs)	0.25 nM	-	No non-specific interaction of antibody with nanodiscs.
1 nM (EGFR nanodiscs)	0.25 nM	3.4%	Antibody is bound to pY1068.

Supplementary Table 1. FCS experiments of EGFR phosphorylation. The Y1068 antibody, which binds specifically to the phosphorylated tyrosine pY1068, was mixed with EGFR labelled with snap surface 594 in nanodiscs. FCS was used to measure the diffusion time of EGFR in nanodiscs via excitation of snap surface 594 (λ_{exc}=550 nm). The diffusion time increased upon addition of the antibody, consistent with an increase in the hydrodynamic radius of EGFR due to the antibody binding to the phosphotyrosines. FCS was also used to measure the diffusion time of an alexa 647 labelled Y1068 antibody (λ_{exc}=640 nm). The diffusion time was initially measured in the absence and presence of empty nanodiscs to test for non-specific interactions with the nanodiscs. The same value was extracted (621 μs) for both measurements. Upon the addition of EGFR nanodiscs, the diffusion time increased (643 μs), again consistent with binding of the antibody to the phosphotyrosines of EGFR. The binding of Y1068 to labelled EGFR in nanodiscs strongly implies the constructs retain phosphorylation functionality.

(EGFR:Y1068 antibody)	Donor lifetime (ns)	Decrease in donor lifetime
1:0	3.08	-
2:1	2.87	6.8%
1:2	2.68	12.9%
1:20	1.61	47.8%

Supplementary Table 2. FRET measurements of EGFR phosphorylation. Alexa 647 labelled Y1068 antibody, which binds specifically to the phosphorylated tyrosine pY1068, was mixed with EGFR labelled with snap surface 594 in nanodiscs. The lifetime of the donor (snap surface 594) was measured using ensemble time-correlated single photon counting as a function of concentration of the acceptor-labeled (alexa647) Y1068 antibody. The donor lifetime decreased as the antibody concentration increased, which was assigned to an increase in energy transfer from the donor to the acceptor dye. The presence of energy transfer indicated antibody binding to EGFR, because the 8.1 nm Förster radius requires the dye pair be in close proximity. These results are consistent with the FCS measurements (Supplementary Table 1), and also strongly imply the constructs retain phosphorylation functionality.

Experiments	Experiment distance (nm)	Simulation distance (nm)
Neutral DMPC bilayer		
WT EGFR, -ligand	11.39 [11.14, 11.65]	9.22 [8.56, 10.56]
WT EGFR, +EGF	8.14 [8.10, 8.22]	7.49 [6.83, 7.83]
WT EGFR, +Cetuximab, +EGF	12.02 [11.60, 12.46]	-
WT EGFR, +EPGN	8.00 [7.92, 8.18]	-
JMneu EGFR, -EGF	10.99 [10.82, 11.12]	-
JMneu EGFR, +EGF	10.76 [10.53, 11.06]	-
CTTneu EGFR, -EGF	12.06 [11.58, 12.43]	-
CTTneu EGFR, +EGF	10.97 [10.74, 11.25]	-
30% anionic POPC-POPS bilayer		
WT EGFR, -ligand	10.10 [9.93, 10.33]	7.38 [6.71, 7.71]
WT EGFR, +EGF	*15.22 [13.91, 17.87]	8.97 [8.30, 10.30]
WT EGFR, +Cetuximab, +EGF	7.98 [7.90, 8.14]	-
WT EGFR, +EPGN	8.00 [8.55, 8.94]	-
JMneu EGFR, -ligand	12.34 [11.90, 12.80]	-
JMneu EGFR, +EGF	11.90 [10.95, 13.12]	-
JMneu EGFR, +EPGN	11.19 [10.64, 12.13]	-
CTTneu EGFR, -EGF	8.45 [8.17, 9.11]	-
CTTneu EGFR, +EGF	8.00 [7.90, 8.14]	-

Supplementary Table 3. Median distances between the membrane and C-terminal end of the protein from smFRET experiments and simulations. The distance values were extracted from the distributions shown in Figs. 2, 3 in the main text and Supplementary Figures 12, 13. The numbers in parenthesis indicates the 95% confidence interval for experiments and the minimal and maximal median value from three equal partitions of data for simulations. Asterisk (*) indicates distance was beyond the FRET range for the snap surface 594 and cy5 dye pair (Ro = 8.4 nm).[8] Source data are provided as a Source Data file.

Time (minutes)	Reduction in phosphorylation (EGF)	Reduction in phosphorylation (Epigen)
0	38%	38%
5	29%	32.4%
15	46.9%	39.4%
30	46.8%	47.4%

Supplementary Table 4. Reduction in EGFR phosphorylation of JMneu EGFR. The level of receptor phosphorylation at tyrosines was quantified from the Western blots shown in Figure 5a. Phosphorylation was reduced for JMneu EGFR compared to WT EGFR in CHO cells upon EGF and epigen stimulation. Source data are provided as a Source Data file.

Time (minutes)	Reduction in phosphorylation
0	56%
5	40.1%
15	49.4%
30	61.9%

Supplementary Table 5. Reduction in EGFR phosphorylation of JMneu EGFR. The level of receptor phosphorylation at Y1068 was quantified from the Western blots shown in Supplementary Figure 24a. Phosphorylation was reduced for JMneu EGFR compared to WT EGFR in CHO cells.

Experiments	Average	Standard deviation	Standard Error of Mean
WT EGFR			
no ligand	1	0	0
+EGF, 5 minutes	2.00	0.32	0.12
+EGF, 15 minutes	2.11	0.85	0.32
+EGF, 30 minutes	2.07	0.93	0.35
+Epigen, 5 minutes	1.11	0.22	0.08
+Epigen, 15 minutes	1.04	0.26	0.10
+Epigen, 30 minutes	0.97	0.18	0.07
JMneu EGFR			
no ligand	0.62	0.15	0.59
+EGF, 5 minutes	1.42	0.83	0.32
+EGF, 15 minutes	1.12	0.36	0.14
+EGF, 30 minutes	1.10	0.24	0.09
+Epigen, 5 minutes	0.75	0.14	0.05
+Epigen, 15 minutes	0.63	0.11	0.04
+Epigen, 30 minutes	0.51	0.15	0.06

Supplementary Table 6. Statistics from Western blot experiments on WT EGFR and JMneu EGFR in the absence and presence of EGF and Epigen. The statistics were quantified from western blot experiments from seven independent biological replicates (Figure 5a, b). Source data are provided as a Source Data file.

Experiment 1	Experiment 2	P-value
WT EGFR, +EGF, 5 minutes	JMneu EGFR, +EGF, 5 minutes	0.1508
WT EGFR, +EGF, 15 minutes	JMneu EGFR, +EGF, 15 minutes	0.0303
WT EGFR, +EGF, 30 minutes	JMneu EGFR, +EGF, 30 minutes	0.0442
WT EGFR, +Epigen, 5 minutes	JMneu EGFR, +Epigen, 5 minutes	0.0069
WT EGFR, +Epigen, 15 minutes	JMneu EGFR, +Epigen, 15 minutes	0.0072
WT EGFR, +Epigen, 30 minutes	JMneu EGFR, +Epigen, 30 minutes	0.0005

Supplementary Table 7. Statistical analysis of western blot distributions. Results from two-tailed, nonparametric, paired t-test for all experimental pairs (experiment 1 and experiment 2 in the above table) from the distributions reported in Figure 5b. Source data are provided as a Source Data file.

Antibody name	Target	Host	Company	Catalog Number	Dilution
Anti-EGFR Antibody (A-10)	EGFR C-terminal	$MouseIgG_{2a}$	Santa Cruz Biotechnology	sc-373746	1:200
Human Phospho-EGFR Y1068 Antibody	EGFR phosphorylated at Y1068	$MouseIgG_{2a}$	R&D Systems	MAB3570	1:200
Goat anti-Mouse IgG (H+L) Highly Cross-Adsorbed Antibody Alexa Fluor 790	Mouse	GoatIgG	Thermo Fisher	A11357	1:10000

Supplementary Table 8. List of antibodies used to show phosphorylation of EGFR nanodiscs produced via cell-free reaction.

Variable	Fit value	95% confidence interval
a	41.8	[10.9, 72.7]
b	1.6	[0.3, 2.9]

Supplementary Table 9. Intracellular conformational compaction decreased by electrostatic screening. Parameters from the fit of the percent change in hydrodynamic radius as a function of salt concentration (Fig. 3b in the main text) using Debye-Huckel theory for electrostatic screening.[9] The goodness of fit (R-square) is 0.95. Source data are provided as a Source Data file.

Experiment 1	Experiment 2	P-value	F	Degrees of freedom
DMPC membrane				
WT EGFR, -ligand	WT EGFR, +EGF	$1.55e^{-154}$	911.3	1398
WT EGFR, +EGF	WT EGFR, +EGF, +Cetuximab	$3.95e^{-126}$	723.2	1254
WT EGFR, -ligand	WT EGFR, +EPGN	$1.98e^{-160}$	541.6	934
JMneu EGFR, -EGF	JMneu EGFR, +EGF	0.0794	3.08	989
CTTneu EGFR, -EGF	CTTneu EGFR, +EGF	0.2579	1.29	139
30% anionic POPC-POPS membrane				
WT EGFR, -ligand	WT EGFR, +EGF	$1.63e^{-37}$	172.1	1695
WT EGFR, +EGF	WT EGFR, +EGF, +Cetuximab	$2.64e^{-75}$	383.1	1352
WT EGFR, -ligand	WT EGFR, +EPGN	$1.79e^{-25}$	113.6	1294
JMneu EGFR, -ligand	JMneu EGFR, +EGF	0.3636	0.83	1043
JMneu EGFR, -ligand	JMneu EGFR, +EPGN	0.0135	6.11	1427
CTTneu EGFR, -EGF	CTTneu EGFR, +EGF	0.7977	0.07	1758

Supplementary Table 10. Statistical analysis of smFRET lifetime distributions. Results from one-way analysis of variance (ANOVA) with P-value, F-statistic and degrees of freedom for all experimental pairs (experiment 1 and experiment 2 in the above table) from the distributions reported in Figure 2, 3 and Supplementary Figures 12, 13. Source data are provided as a Source Data file.

Experiments	Median (ns)	Max (ns)	Min (ns)	Whiskers (ns)	Quartiles (ns)
Neutral DMPC bilayer					
WT EGFR, -ligand	2.88	4.80	0.83	[1.18, 4.50]	[2.43, 3.29]
WT EGFR, +EGF	1.51	3.74	0.66	[0.66, 3.68]	[1.31, 2.38]
WT EGFR, +Cetuximab, +EGF	2.99	4.65	0.89	[1.15, 4.65]	[2.50, 3.41]
WT EGFR, +neuregulin	2.92	4.78	1.00	[1.48, 4.33]	[2.54, 3.27]
WT EGFR, +epigen	1.42	3.48	0.34	[0.34, 3.48]	[1.13, 2.14]
WT EGFR, donor only	3.34	5.00	1.27	[1.74, 5.00]	[2.94, 3.77]
JMneu EGFR, -EGF	2.14	3.58	0.91	[1.28, 2.96]	[1.92, 2.35]
JMneu EGFR, +EGF	2.10	3.90	0.59	[0.75, 3.29]	[1.73, 2.41]
JMneu EGFR, donor only	2.57	4.29	1.13	[1.28, 3.59]	[2.18, 2.81]
CTTneu EGFR, -EGF	2.43	4.01	1.23	[1.23, 3.63]	[2.10, 2.74]
CTTneu EGFR, +EGF	2.26	3.93	0.91	[1.05, 3.24]	[1.94, 2.54]
CTTneu EGFR, donor only	2.71	4.93	1.60	[1.68, 3.74]	[2.45, 2.97]

Supplementary Table 11. Statistics from single-molecule measurements in neutral DMPC lipid bilayer. The median, maximum, minimum, whiskers and quartiles for experimental data in Figure 2a, Supplementary Figures 7, 8, 12, 13. Source data are provided as a Source Data file.

Experiments	Median (ns)	Max (ns)	Min (ns)	Whiskers (ns)	Quartiles (ns)
30% anionic POPC-POPS bilayer					
WT EGFR, -ligand	1.93	3.60	0.31	[0.31, 3.60]	[1.48, 2.54]
WT EGFR, +EGF	2.50	5.00	0.55	[0.83, 4.02]	[2.03, 2.83]
WT EGFR, +Cetuximab, +EGF	1.12	4.36	0.50	[0.50, 4.36]	[0.93, 2.36]
WT EGFR, +epigen	1.48	3.00	0.28	[0.28, 3.00]	[1.03, 1.99]
WT EGFR, donor only	2.57	4.95	0.65	[1.05, 4.07]	[2.19, 2.95]
JMneu EGFR, -EGF	2.38	3.99	0.70	[0.70, 3.73]	[1.91, 2.73]
JMneu EGFR, +EGF	2.33	3.50	0.82	[0.82, 3.50]	[1.80, 2.73]
JMneu EGFR, donor only	2.62	5.00	0.87	[0.87, 4.33]	[2.16, 3.04]
CTTneu EGFR, -EGF	1.39	4.50	0.67	[0.67, 4.50]	[0.94, 2.41]
CTTneu EGFR, +EGF	1.17	5.00	0.52	[0.52, 4.39]	[0.95, 2.38]
JMneu EGFR, donor only	2.73	5.00	1.18	[1.45, 4.04]	[2.41, 3.07]

Supplementary Table 12. Statistics from single-molecule measurements in partially anionic POPC-POPS lipid bilayer. The median, maximum, minimum, whiskers and quartiles for experimental data in Figure 2d, 3c, Supplementary Figures 9, 13. Source data are provided as a Source Data file.

Experiments	Number of molecules	Number of bunches
Neutral DMPC bilayer		
WT EGFR, -ligand	51	702
WT EGFR, +EGF	53	697
WT EGFR, +Cetuximab, +EGF	61	558
WT EGFR, +EPGN	44	233
JMneu EGFR, -EGF	48	563
JMneu EGFR, +EGF	66	427
CTTneu EGFR, -EGF	59	418
CTTneu EGFR, +EGF	77	567
30% anionic POPC-POPS bilayer		
WT EGFR, -ligand	128	891
WT EGFR, +EGF	121	805
WT EGFR, +Cetuximab, +EGF	65	548
WT EGFR, +EPGN	125	404
JMneu EGFR, -ligand	96	809
JMneu EGFR, +EGF	66	235
JMneu EGFR, +EPGN	93	619
CTTneu EGFR, -EGF	51	965
CTTneu EGFR, +EGF	69	794

Supplementary Table 13. Sample sizes for smFRET measurements. The number of molecules and number of photon bunches used to construct the lifetime distributions are reported for all smFRET histograms from Figure 2, 3 in the main text and Supplementary Figure 12, 13.

Experiment 1	Experiment 2	P-value
WT EGFR, -EGF	JMneu EGFR, -EGF	0.002
WT EGFR, +EGF, 5 minutes	JMneu EGFR, +EGF, 5 minutes	0.102
WT EGFR, +EGF, 15 minutes	JMneu EGFR, +EGF, 15 minutes	0.033
WT EGFR, +EGF, 30 minutes	JMneu EGFR, +EGF, 30 minutes	0.049

Supplementary Table 14. Statistical analysis of western blot distributions. Results from two-tailed, non-parametric, paired t-test for all experimental pairs (experiment 1 and experiment 2 in the above table) from the distributions reported in Supplementary Figure 24 a, b. Source data are provided as a Source Data file.

Experiments	Average	Standard deviation	Standard Error of Mean
WT EGFR			
no ligand	1	0	0
+EGF, 5 minutes	3.75	1.53	0.63
+EGF, 15 minutes	3.12	1.16	0.48
+EGF, 30 minutes	3.23	1.73	0.70
JMneu EGFR			
no ligand	0.45	0.22	0.09
+EGF, 5 minutes	2.24	1.04	0.43
+EGF, 15 minutes	1.58	0.69	0.28
+EGF, 30 minutes	1.23	0.41	0.17

Supplementary Table 15. Statistics from Western blot experiments on WT EGFR and JMneu EGFR. The statistics were quantified from western blot experiments from six independent biological replicates in Supplementary Figure 24 a, b. Source data are provided as a Source Data file.

Antibody name	Species	Company	Catalog Number	Dilution
EGFR	Rabbit	Cell Signaling Technologies	2232	1:1000
Phospho-Tyrosine (P-Tyr-1000)	Rabbit	Cell Signaling Technologies	8954S	1:2000
Phospho-EGF Receptor (Tyr1068)	Rabbit	Cell Signaling Technologies	2234	1:1000
Phospho-EGF Receptor (Tyr1045)	Rabbit	Cell Signaling Technologies	2237	1:1000
Phospho-EGF Receptor (Tyr992)	Rabbit	Cell Signaling Technologies	2235	1:1000
Akt	Rabbit	Cell Signaling Technologies	9272	1:1000
Phospho-Akt (Ser473)	Rabbit	Cell Signaling Technologies	9271	1:1000
p44/42 MAPK (Erk1/2)	Rabbit	Cell Signaling Technologies	9102	1:1000
Phospho-p44/42 MAPK (Erk1/2) (Thr202/Tyr204)	Rabbit	Cell Signaling Technologies	9101	1:1000
β-Actin	Mouse	Sigma	A1978	1:10000
Goat Anti-Mouse IgG (H+L)-HRP Conjugate	Goat	Bio-Rad	1706516	1:5000
Goat Anti-Rabbit IgG (H+L)-HRP Conjugate	Goat	Bio-Rad	1706515	1:5000

Supplementary Table 16. List of antibodies used in cell phosphorylation experiments.

Experiments	Experiment distance (nm)	Simulation distance (nm)
Neutral DMPC membrane		
WT EGFR, -EGF	7.97 [7.83, 8.13]	7.00 [6.00, 8.00]
WT EGFR, +EGF	8.46 [8.28, 8.57]	5.84 [5.17, 7.17]
30% anionic POPC-POPS membrane		
WT EGFR, -EGF	9.03 [8.93, 9.17]	5.66 [5.66, 5.66]
WT EGFR, +EGF	10.89 [10.50, 11.54]	7.50 [6.17, 9.17]

Supplementary Table 17. Median distances between the ATP binding site (residue 721) and the C-terminal end of the protein (residue 1186) from smFRET experiments and simulations. The distance values were extracted as the medians of the distributions (Supplementary Figure 17), which show that the simulations replicate the experimental trends. The numbers in parenthesis indicates the 95% confidence interval for experiments and the minimal and maximal median value from three equal partitions of data for simulations. Source data are provided as a Source Data file.

Supplementary References

[1] He, W. *et al.* Cell-free expression of functional receptor tyrosine kinases. *Scientific reports* **5**, 12896–12903 (2015).

[2] Quinn, S. D. *et al.* Single-Molecule Fluorescence Detection of the Epidermal Growth Factor Receptor in Membrane Discs. *Biochemistry* **58**, 286–294 (2018).

[3] Britsch, S. The neuregulin-I/ErbB signaling system in development and disease (2007).

[4] Hawe, A., Sutter, M. & Jiskoot, W. Extrinsic fluorescent dyes as tools for protein characterization. *Pharmaceutical research* **25**, 1487–99 (2008).

[5] Jo, S., Kim, T., Iyer, V. G. & Im, W. CHARMM-GUI: a web-based graphical user interface for CHARMM. *Journal of computational chemistry* **29**, 1859–1865 (2008).

[6] Fiser, A., Do, R. K. G. & ali, A. Modeling of loops in protein structures. *Protein science*, **9**, 1753–177 (2000).

[7] Arkhipov, A *et al.* & Shaw, D. E. Architecture and membrane interactions of the EGF receptor. *Cell* **152**, 557–569 (2013).

[8] Sisamakis, E., Valeri, A., Kalinin, S., Rothwell, P. J. & Seidel, C. A. Accurate single-molecule FRET studies using multiparameter fluorescence detection. *Methods in enzymology* **475**, 455–514 (2010).

[9] Tsai, M. Y. *et al.* Electrostatics, structure prediction, and the energy landscapes for protein folding and binding. *Protein Science* **25** 255–269 (2016).
