# Supplementary material for: Ligand-induced transmembrane conformational coupling in monomeric EGFR
Source: Nat Commun. 2022 Jul 6;13:3709. doi: 10.1038/s41467-022-31299-z (PMC9259572; doi:10.1038/s41467-022-31299-z)
Supplement: Supplementary file 3 — Reporting Summary [file 41467_2022_31299_MOESM3_ESM.pdf]

nature research

Corresponding author(s): Gabriela Schlau-Cohen, Bin Zhang

Last updated by author(s): Jun 2, 2022

Reporting Summary

Nature Research wishes to improve the reproducibility of the work that we publish. This form provides structure for consistency and transparency in reporting. For further information on Nature Research policies, see our Editorial Policies and the Editorial Policy Checklist.

Statistics

For all statistical analyses, confirm that the following items are present in the figure legend, table legend, main text, or Methods section.

n/a	Confirmed	
[ ]	[x]	The exact sample size (n) for each experimental group/condition, given as a discrete number and unit of measurement
[ ]	[x]	A statement on whether measurements were taken from distinct samples or whether the same sample was measured repeatedly
[ ]	[x]	The statistical test(s) used AND whether they are one- or two-sided<br>*Only common tests should be described solely by name; describe more complex techniques in the Methods section.*
[x]	[ ]	A description of all covariates tested
[x]	[ ]	A description of any assumptions or corrections, such as tests of normality and adjustment for multiple comparisons
[ ]	[x]	A full description of the statistical parameters including central tendency (e.g. means) or other basic estimates (e.g. regression coefficient) AND variation (e.g. standard deviation) or associated estimates of uncertainty (e.g. confidence intervals)
[ ]	[x]	For null hypothesis testing, the test statistic (e.g. F, t, r) with confidence intervals, effect sizes, degrees of freedom and P value noted<br>*Give P values as exact values whenever suitable.*
[x]	[ ]	For Bayesian analysis, information on the choice of priors and Markov chain Monte Carlo settings
[x]	[ ]	For hierarchical and complex designs, identification of the appropriate level for tests and full reporting of outcomes
[x]	[ ]	Estimates of effect sizes (e.g. Cohen's d, Pearson's r), indicating how they were calculated

Our web collection on statistics for biologists contains articles on many of the points above.

Software and code

Policy information about availability of computer code

Data collection	LabVIEW (version 2018-2021) was used for data collection for this work and is commercially available.
Data analysis	LabVIEW (version 2018-2021) was used for data collection for this work and is commercially available.<br>The data analysis for the single-molecule data was done using MATLAB (version 9.8-9.11) . The MATLAB code is available from the corresponding authors upon request.

For manuscripts utilizing custom algorithms or software that are central to the research but not yet described in published literature, software must be made available to editors and reviewers. We strongly encourage code deposition in a community repository (e.g. GitHub). See the Nature Research guidelines for submitting code & software for further information.

Data

Policy information about availability of data

All manuscripts must include a data availability statement. This statement should provide the following information, where applicable:

- Accession codes, unique identifiers, or web links for publicly available datasets
- A list of figures that have associated raw data
- A description of any restrictions on data availability

The source data that support the findings of the present study are provided with this paper. The molecular dynamics simulation data generated in this study have been deposited in the zenodo database (DOI: 10.5281/zenodo.6564353). The following PDB files were used for the construction of the monomer for the MD simulations: 3NJP [http://doi.org/10.2210/pdb3NJP/pdb], 2GS6 [http://doi.org/10.2210/pdb2GS6/pdb], 2JWA [http://doi.org/10.2210/pdb2JWA/pdb], [http://doi.org/10.2210/pdb3GOP/pdb], 1NQL [http://doi.org/10.2210/pdb1NQL/pdb], 3GT8 [http://doi.org/10.2210/pdb3GT8/pdb].

Field-specific reporting

Please select the one below that is the best fit for your research. If you are not sure, read the appropriate sections before making your selection.

- [x] Life sciences
- [ ] Behavioural & social sciences
- [ ] Ecological, evolutionary & environmental sciences

For a reference copy of the document with all sections, see nature.com/documents/nr-reporting-summary-flat.pdf

Life sciences study design

All studies must disclose on these points even when the disclosure is negative.

Sample size	The sample sizes for the single-molecule experiments are provided in the supplementary information and was selected based on other immobilization based single-molecule FRET experiments on a confocal setup (PNAS, 97, 13021, 2000; PNAS, 94, 7932, 1997). No sample size calculations were performed for the in vivo experiments. The number of biological replicates performed was to ensure statistically significant differences and draw valid conclusions. At least three independent experiments were carried out as has been historically accepted.
Data exclusions	There were no data exclusions in our study.
Replication	Replicate information for the single-molecule FRET and in vivo experiments can be found in the respective figure legends.
Randomization	This information does not apply to our submission: no experimental groups or group allocation were performed or designed.
Blinding	Blinding was not applicable in these contexts because group allocation was not required for these studies.

Reporting for specific materials, systems and methods

We require information from authors about some types of materials, experimental systems and methods used in many studies. Here, indicate whether each material, system or method listed is relevant to your study. If you are not sure if a list item applies to your research, read the appropriate section before selecting a response.

Materials & experimental systems

n/a	Involved in the study	
[ ]	[x]	Antibodies
[ ]	[x]	Eukaryotic cell lines
[x]	[ ]	Palaeontology and archaeology
[x]	[ ]	Animals and other organisms
[x]	[ ]	Human research participants
[x]	[ ]	Clinical data
[x]	[ ]	Dual use research of concern

Methods

n/a	Involved in the study	
[x]	[ ]	ChIP-seq
[x]	[ ]	Flow cytometry
[x]	[ ]	MRI-based neuroimaging

Antibodies

Antibodies used

1. Anti-EGFR Antibody (A-10) from Santa Cruz BioTechnology (Catalog Number: sc-373746)
2. Human Phospho-EGFR (Y1068) Antibody from R&D systems (Catalog Number: MAB3570)
3. Goat anti-Mouse IgG (H+L) Highly Cross-Adsorbed Antibody Alexa Fluor 790 from ThermoFisher (Catalog Number: A11357)
4. EGFR from Cell Signaling Technologies (Catalog Number: 2232)
5. Phospho-Tyrosine (P-Tyr-1000) from Cell Signaling Technologies (Catalog Number: 8954S)
6. Phospho-EGF Receptor (Tyr1068) from Cell Signaling Technologies (Catalog Number: 2234)
7. Phospho-EGF Receptor (Tyr1045) from Cell Signaling Technologies (Catalog Number: 2237)
8. Phospho-EGF Receptor (Tyr992) from Cell Signaling Technologies (Catalog Number: 2235)
9. Akt from Cell Signaling Technologies (Catalog Number: 9272)
10. Phospho-Akt (Ser473) from Cell Signaling Technologies (Catalog Number: 9271)
11. p44/42 MAPK (Erk1/2) from Cell Signaling Technologies (Catalog Number: 9102)
12. Phospho-p44/42 MAPK (Erk1/2) (Thr202/Tyr204) from Cell Signaling Technologies (Catalog Number: 9101)
13. β-Actin from Sigma (Catalog Number: A1978)
14. Goat Anti-Mouse IgG (H + L)-HRP Conjugate from BioRad (Catalog Number: 1706516)
15. Goat Anti-Rabbit IgG (H + L)-HRP Conjugate from BioRad (Catalog Number: 1706515)

Validation

Antibodies 1-3 from above were also confirmed by our lab using cell-free experiments. Anti-EGFR Antibody (A-10) from Santa Cruz BioTechnology (Catalog Number: sc-373746) has been used for detection of EGFR in cell lysates (Mol Cancer Ther., 20(9), 1640, 2021; Cell Reports, 37, 110096, 2021). Human Phospho-EGFR (Y1068) Antibody from R&D systems (Catalog Number: MAB3570) has been used for detection of phosphorylated Y1068 in EGFR in cell lysates (Translational Oncology, 14(2), 100961, 2020; Nat Commun, 10

(1), 909, 2019). Antibodies 4-15 from above are all commonly used and commercially available. The antibodies used were validated by the manufacturers for their respective applications. This information is provided in the antibody datasheets or on the manufacturers' websites. https://www.cellsignal.com/about-us/cst-antibody-validation-principles.

Eukaryotic cell lines

Policy information about cell lines

Cell line source(s)	CHO cells were purchased from American Type Culture Collection (ATCC)
Authentication	The cell lines were authenticated by comparing the STR profile of sample cell lines with the ATCC Human Cell STR Database
Mycoplasma contamination	The cell line tested negative for mycoplasma contamination. ATCC uses a proprietary PCR-based mycoplasma assay to test cell lines
Commonly misidentified lines (See ICLAC register)	None
